# Supplementary material for: Estimating global identifiability using conditional mutual information in a Bayesian framework
Source: Sci Rep. 2023 Oct 26;13:18336. doi: 10.1038/s41598-023-44589-3 (PMC10603099; doi:10.1038/s41598-023-44589-3)
Supplement: Supplementary file 1 — Supplementary Information. [file 41598_2023_44589_MOESM1_ESM.pdf]

Supplementary Material: Estimating global identifiability using conditional mutual information in a Bayesian framework

Sahil Bhola[1*] and Karthik Duraisamy[1]

[1]Department of Aerospace Engineering, University of Michigan, Ann Arbor, 48109, Michigan, USA.

*Corresponding author(s). E-mail(s): sbhola@umich.edu;
Contributing authors: kdur@umich.edu;

S1 Global Sobol Sensitivity Analysis

Variance-based sensitivity analysis has been widely used to determine the most influential parameters by examining variability in the forward model response as a result of uncertainty in the model parameters [29]. This is primarily based on the idea that parameters which cause large variability in model output are most influential and therefore relevant in the context of parameter estimation. Note that while sensitivity analysis comments on the degree of variability in the model output due to parameter uncertainty, it does not quantify the degree of confidence in parameter estimation and therefore practical identifiability. While parameter sensitivity is necessary for identifiability it is not sufficient [31].

Global sensitivity analysis based on Sobol indices apportions the variability in the response of the forward model only with respect to uncertainty in the parameters. Consider the statistical model in (1) with no measurement uncertainty, that is $y = \mathscr{F}(\theta, d)$. Without the loss of generality, consider the model output as a scalar. For such a model the total output variability can be uniquely decomposed as

$$\mathbb{V}\text{ar}_{\Theta}[Y] \triangleq \sum_{i=1}^{m} V_i + \sum_{i<j}^{m} V_{ij} + \cdots + V_{12\ldots m}, \tag{S1}$$

under the assumption that all $\Theta_i \in \Theta$ are mutually statistically independent [13]. Here

$$V_i \triangleq \mathbb{V}\mathrm{ar}_{\Theta_i}[\mathbb{E}_{\Theta_{\sim i}}[Y \mid \Theta_i]], \tag{S2}$$

$$V_{ij} \triangleq \mathbb{V}\mathrm{ar}_{\Theta_i,\Theta_j}[\mathbb{E}_{\Theta_{\sim i,j}}[Y \mid \Theta_i, \Theta_j]] - V_i - V_j, \tag{S3}$$

and so forth. Variance-based Sobol indices can then be defined as

$$S_i \triangleq \frac{V_i}{\mathbb{V}\mathrm{ar}_{\Theta}[Y]}, \tag{S4}$$

$$S_{ij} \triangleq \frac{V_{ij}}{\mathbb{V}\mathrm{ar}_{\Theta}[Y]}, \tag{S5}$$

and so forth. As a result

$$\sum_{i=1}^{m} S_i + \sum_{i<j}^{m} S_{ij} + \cdots + S_{12\ldots m} = 1. \tag{S6}$$

The S_i terms are called the first-order or the main effect Sobol indices, which apportions the output variability due to uncertainty in Θ_i. The multi-index terms such as S_{ij} are the higher order interaction terms that apportion the output variability due to the combined uncertainty in parameter sets. For example, the second-order Sobol indices S_{ij} apportions the output variability due to the interaction between parameter pairs. Note, in the case of vector model output the expected Sobol indices can be evaluated. In most practical applications, the first-order Sobol indices in (S4) are sufficient to ascertain parameter relevance from the perspective of model sensitivity. In the present work, the Sobol indices are estimated using `SALib` [42, 43]. It requires $N*(2m+2)$ model evaluations, where N is the number of parameter samples generated and m is the number of parameters in the statistical model.

S2 Linear Gaussian Model

For a random variable Z distributed normally as $\mathcal{N}(\mu, \Sigma)$ the differential entropy has a closed form expression

$$H(Z) = \frac{n}{2}\ln(2\pi) + \frac{1}{2}\ln|\Sigma| + \frac{1}{2}n, \tag{S7}$$

where $\mu \in \mathbb{R}^n$ and $\Sigma \in \mathbb{R}^{n\times n}$ [38].

Consider the differential entropy formulation for information gain in (9b). To evaluate information gain for a linear Gaussian model in (26), closed-form expressions are available for the densities $p(\theta_i, \theta_{\sim i} \mid d)$, $p(\theta_{\sim i}, y \mid d)$, $p(\theta_{\sim i} \mid d)$, and $p(\theta_i, \theta_{\sim i|d}, y \mid d)$ such that (S7) can be used to find the true information gain.

Similarly, consider the differential form for evaluating the parameter dependencies in (22b). Closed form expressions are available for the densities $p(\theta_i, \theta_{\sim i,j}, y \mid d)$, $p(\theta_j, \theta_{\sim i,j}, y \mid d)$, $p(\theta_{\sim i,j}, y \mid d)$, and $p(\theta_i, \theta_j \theta_{\sim i,j} \mid d)$ such that true parameter dependencies can be evaluated using (S7).

S3 Convergence of Sobol Indices

S3.1 Linear Gaussian Model

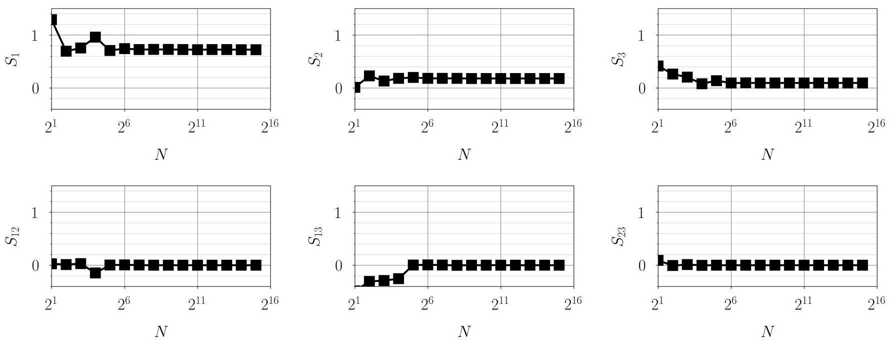

Fig. S1 Convergence of first-order Sobol indices (top) and second-order Sobol indices (bottom) with number of parameter samples N (see §S1) for linear Gaussian model.

S3.2 Methane Chemical Kinetics Model

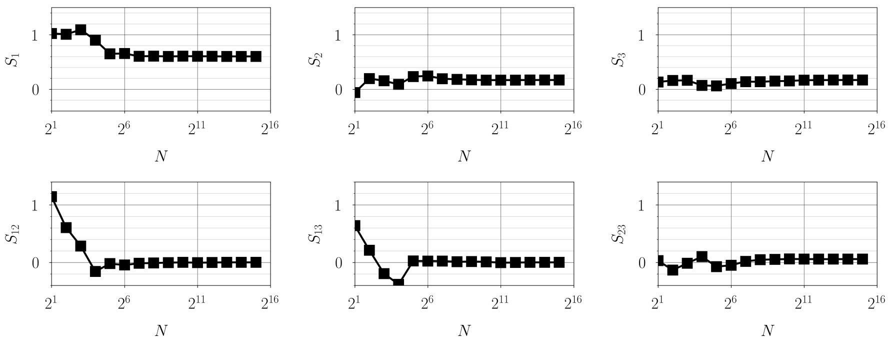

Fig. S2 Convergence of first-order Sobol indices (top) and second-order Sobol indices (bottom) with number of parameter samples N (see §S1) for methane-air combustion model.
